# Phenological growth stages of Finger millet (*Eleusine coracana* (L.)) using 3-D phenotyping with relevance to breeding applications

**DOI:** 10.3389/fpls.2026.1747707

**Published:** 2026-07-28

**Authors:** Juan I. Di Salvo, Shambhavi Joshi, Mozhgan Hadadi, Talukder Jubery, Soumik Sarkar, Arti Singh, Baskar Ganapathysubramanian, Sotirios Archontoulis, Adarsh Krishnamurthy, Asheesh K. Singh

**Affiliations:** 1 Department of Agronomy, Iowa State University, Ames, IA, United States; 2 Department of Mechanical Engineering, Iowa State University, Ames, IA, United States

**Keywords:** 3-D (three-dimension), Finger millet (Eleusine coracana), neural radiance fields (NeRF), phenology, virtual reality

## Abstract

Finger millet (*Eleusine coracana* (L.) Gaertn.) is a small seeded cereal known for its health benefits and its ability to grow in water-limited and saline environments. Despite its health and adaptation advantages, there is no standardized BBCH scale developed for finger millet, precluding standardization of a defined growth and development scale. The initial step in any breeding program and related research, is the precise, consistent and standardized description of crop growth stages. Therefore, we present a standardized phenological scale to describe and compare different stages of growth and development. In this study, we used four-finger millet accessions from the U.S.D.A. National Plant Germplasm System (NPGS) collection. Using a standardized BBCH chart as the baseline, we describe nine main stages of development to provide a simplified and user-friendly phenological growth staging scale for finger millet. A novel feature of this study was the use of Neural Radiance Fields (NeRF) to generate three-dimensional (3-D) renderings that align growth stages with 3-D imaging, paving the way for future phenological staging studies to incorporate 3-D visualization as a tool for standardization, researcher training, and reduction of observer bias across environments.

## 1 Introduction

Millets are a group of small-seeded grasses (for example*, Eleusine coracana, Echinochloa* spp.*, Setaria italica, Panicum miliaceum*, among others) that have been grown for centuries in semi-arid regions of Africa and Asia (Hilu and Wet, 1976). Despite their resilience and nutritional value, these crops have been largely displaced by major cereal crops, such as maize (*Zea mays* L.), wheat (*Triticum aestivum* L.), and rice (*Oryza sativa* L.) which receive more attention in global agriculture (Meena et al., 2021; Singh et al., 2021a).These species generally produce reasonable yields under low inputs and adverse conditions. With a global production area of approximately 31 million hectares (Food and Organization, 2021), millets are cultivated worldwide. Given their broad adaptability millets are recognized as a strategic crop for addressing the challenges of a highly variable climate (IFAD and UNICEF, 2020; Krishna et al., 2021; Krishnamurthy et al., 2016; Kumar et al., 2018; Meena et al., 2021; Singh et al., 2021a).

Finger millet (*Eleusine coracana* (L.) Gaertn.) is an annual, self-pollinated, small-seeded cereal crop. It was domesticated 5, 000 years ago in eastern Africa and later introduced and domesticated in India, commonly known as Ragi. This annual grass has proven to be a hardy crop that can grow under harsh environmental conditions (drought, salinity, etc.) (Gupta et al., 2017; Singh et al., 2021b). This cereal may be an unknown crop in the western hemisphere, but it has been widely cultivated in some regions of Africa and Asia with grain yield reports ranging from 0.7 to 7.3Mg ha^-^1 (Krishna et al., 2021; Pradhan et al., 2010; Rao et al., 1986; Talwar et al., 2020; Tesfaye et al., 2023; Upadhyaya et al., 2011). There have been advances in finger millet research in the areas of agronomy (Baath et al., 2021; Gowda et al., 2015) quality (Gani and Shinggu, 2023) and genomics (Bančič et al., 2023). Recently the finger millet genome has been sequenced, providing breeders and researchers with new tools (Devos et al., 2023). Finger millet offers a healthy nutritional profile (rich in fiber and are naturally gluten-free) containing high levels of essential micronutrients like calcium, iron and phosphorus and vitamins, making them a valuable food (Sabuz et al. 2023). However, there is still a glaring gap in finger millet research.

A defined growth and development scale has been described for most major crops. For example, soybean (*Glycine max* (L.) Merr.) (Fehr et al., 1971), maize (Hanway, 1966), wheat (Feekes, 1941), sunflower (*Helianthus annuus* L.) (Schneiter and Miller, 1981). However, in finger millet, a standardized developmental stage descriptor is lacking. This deficiency limits the standardization of research and results reporting, hindering breeders and researchers from conducting studies and validating results. It also complicates the comparison of methods for trait analysis and phenotyping.The **B**iologische Bundesanstalt **B**unddessortenamt and **CH**emical industry scale (**BBCH**) provides a standardized system for coding phenologically similar growth stages across all mono- and dicotyledonous plant species. This scale uses a two-digit decimal code, with ten principal growth stages (0-9) and corresponding secondary or sub-stages (0-9), resulting in a comprehensive range from 00 to 99. This scale has been widely applied to describe the growth stages of various mono- and dicotyledonous weeds (Hess et al., 1997) crop species (Archontoulis et al., 2010; Duquette and Kimball, 2020; Sosa-Zuniga et al., 2017) and model plants (Junqueira et al., 2020).

Despite its utility, the exhaustive level of detail of the BBCH scale can be challenging to apply in practice and, in some instance, leads to confusion. Certain stages may be highly relevant to one species but less applicable to others, and the full set of 99 stages can be difficult for observers to track and manage effectively. While standardized definitions are essential for consistency in research and crop management, there is interest in developing a simplified, user-friendly version of the BBCH scale with breeding applications retaining the core principles of the original scale, while focusing on the most relevant stages for physiological studies, cultivar development and practical field applications.

The simplified scale can constantly be updated to reflect the most relevant stages. Moreover, integrating digital imaging technologies can improve the visualization and understanding of the plant development (Martinez-Guanter et al., 2019). Historically, BBCH charts have been supported by illustrations and 2-dimentional (2-D) digital images (Munger et al., 1998), which help users connect phenological data with observable traits.

Advancements in sensor technology now allow for the capture of 3-dimensional (3-D) data, offering a more immersive and interacting approach to crop staging. By converting 2-D images or video into detailed 3-D models (Arshad et al., 2024; Chiranjeevi et al., 2021; Maraveas, 2024; Young et al., 2023), researchers can create virtual environments where users can explore and engage with crop development in real time. These immersive platforms (Ganapathysubramanian et al., 2025; Slob et al., 2023), can provide a richer more informative experience that bridges the gap between abstract growth stages and tangible plant phenotypes. Recent advances have further demonstrated the utility of NeRF-based reconstruction for capturing detailed plant geometry under controlled and field conditions, with multimodal imaging pipelines offering complementary structural information across plant canopies (Arshad et al., 2024; Stumpe et al., 2025). Reviews of 3-D reconstruction methods in agriculture highlight that NeRF occupies a distinct niche among available techniques, offering photorealistic volumetric renders from multi-view imagery particularly suited for visualization and educational applications, albeit at higher computational cost relative to classical Structure-from-Motion approaches (Li et al., 2025; Hu et al., 2024).

This work proposes the development of a reliable, simplified, and standardized phenological scale for finger millet, grounded in the BBCH framework. Unlike traditional scales that rely solely on textual or 2-D descriptions, our approach introduces a novel integration of 3-D imaging and virtual reality environments to visually represent each growth and developmental stage, representing the first integration of NeRF-based 3-D imaging with a standardized phenological staging scale for finger millet.

## 2 Materials and methods

Greenhouse experiments conducted over two consecutive years, 2023 and 2024, with goal of developing a simplified phenological scale for finger millet. The 2023 experiment focused on identifying and documenting key growth stages using 2-D imaging while the 2024 experiment aimed to validate those observations and generate images for the 3-D reconstruction of plant development.

### 2.1 Experiment I (greenhouse 2023)

Two finger millet accessions (PI 462771 and PI 462417) from the U.S.D.A. National Plant Germplasm System (NPGS) collection held at the Plant Genetic Resources Conservation Unit at Griffin, GA, USA, were selected in this study. These genotypes were chosen based on visual assessments and agronomic performance during the 2022 growing season at the Agricultural Engineering/Agronomy (AEA) research farm near Ames, IA (42.01 N, 93.77 W) (Di Salvo, 2024). Selection criteria included mid-maturity lines compatible with Iowa latitudes, erect stem architecture, and robust canopy development.

Greenhouse conditions were maintained at 24.2 ± 1.98 °C with 16-hour photoperiod. Fertilizer and irrigation were applied as needed to ensure optimal growth. Phenological stages were recorded daily during vegetative stages, increasing the time between observations to twice a week after flowering.

Germination staging: A total of nine seeds per accession, arranged as three replicates of three seeds each were surface sterilized with a 10% sodium hypochlorite solution, rinsed with deionized water, and placed in three petri dishes containing soaked filter paper. Dishes were kept in the greenhouse, and germination stages were recorded daily, following the protocol described by Ventura et al. (2020).

Post-germination staging: Seeds of PI 462771 and PI 462417 were planted in 1-gallon Pro-Cal Premium nursery pots filled to ¾ capacity (2800 cm³) with Sun Gro potting mix (S.S. #1 – F1P, Gro Horticulture) and fertilized with 13 g of Osmocote (14-14-14). Each pot received three seeds planted at a depth of 2.5 cm. After emergence, pots were thinned to one plant per unit. Five replicates per accession were arranged in a randomized complete block design (RCBD) to account for spatial variability within the Advanced Teaching Research Building (ATRB) greenhouse at Iowa State University.

#### 2.1.1 Imaging

At each recorded stage images were captured using a Canon EOS Rebel T5i camera. Pots were placed on a short table, with black cloth on the background to enhance contrast. Camera settings were adjusted to produce high-quality JPG/JPEG images (18M, 3840 x 2160 pixels) and white light balance was set to Tungsten light (Approx 3200K) to counteract greenhouse lighting.

### 2.2 Experiment II (greenhouse 2024)

To expand genetic diversity and validate prior observations, two additional accessions—PI 462441 and PI 462765—were selected based on performance in 2023 field trials at Brunner Farm near Ames, IA (42.01°N, 93.72°W) (Di Salvo, 2024). Maturity of the accessions was comparable to that observed in the 2023 experiment. Across both experiments, a total of four accessions were evaluated. Because the study aimed to describe the crop’s most relevant developmental stages rather than quantify the timing of each stage, the four genotypes selected exhibited a similar maturity range. Greenhouse conditions were maintained at 24.6 ± 1.9 °C with a 13-hour photoperiod. Since the base temperature for finger millet was considered 10 °C (Singh et al., 2017), the relatively constant greenhouse temperature of approximately 24 °C resulted in a daily accumulation of about 14 °Cd. This steady accumulation provided a consistent framework for expressing stage progression in thermal time, ensuring that phenological development could be interpreted in terms of physiological thresholds rather than absolute chronology. Five seeds per accession were planted at a depth of 2.5 cm in 4-gallon buckets filled with Sun Gro potting mix (S.S. #1 – F1P, Gro Horticulture) and fertilized with 13 g of Osmocote (14-14-14). After emergence, each bucket was thinned to a single plant. An additional 1 g of urea (46-0-0) was applied three weeks after emergence. Five replicates per genotype were arranged in a completely randomized design (CRD), and plant positions were reassigned biweekly to minimize environmental bias. Phenological observations were conducted three times per week. At each stage, digital photos and 360-degree images were captured to support 3-D reconstruction. Plants were monitored through to maturity, following protocols consistent with the 2023 experiment. The main stem of each plant was identified and tagged, and all vegetative and reproductive stages were recorded on the main stem.

### 2.3 Seed development image acquisition

Approximately 5–7 days after full flowering, 3–5 seeds were carefully extracted from the central portion of a finger on the main stem. The seeds were immediately transported to the laboratory, where they were dissected under a Stereo Microscope to assess internal content. Imaging was performed with an Olympus DP20 microscope camera at each dissection to document the progression of seed development. This procedure was repeated sequentially until the seeds reached full physiological maturity, ensuring a complete visual record of the developmental process.

### 2.4 Reconstruction of digital 3-D models

**Figure 1:**
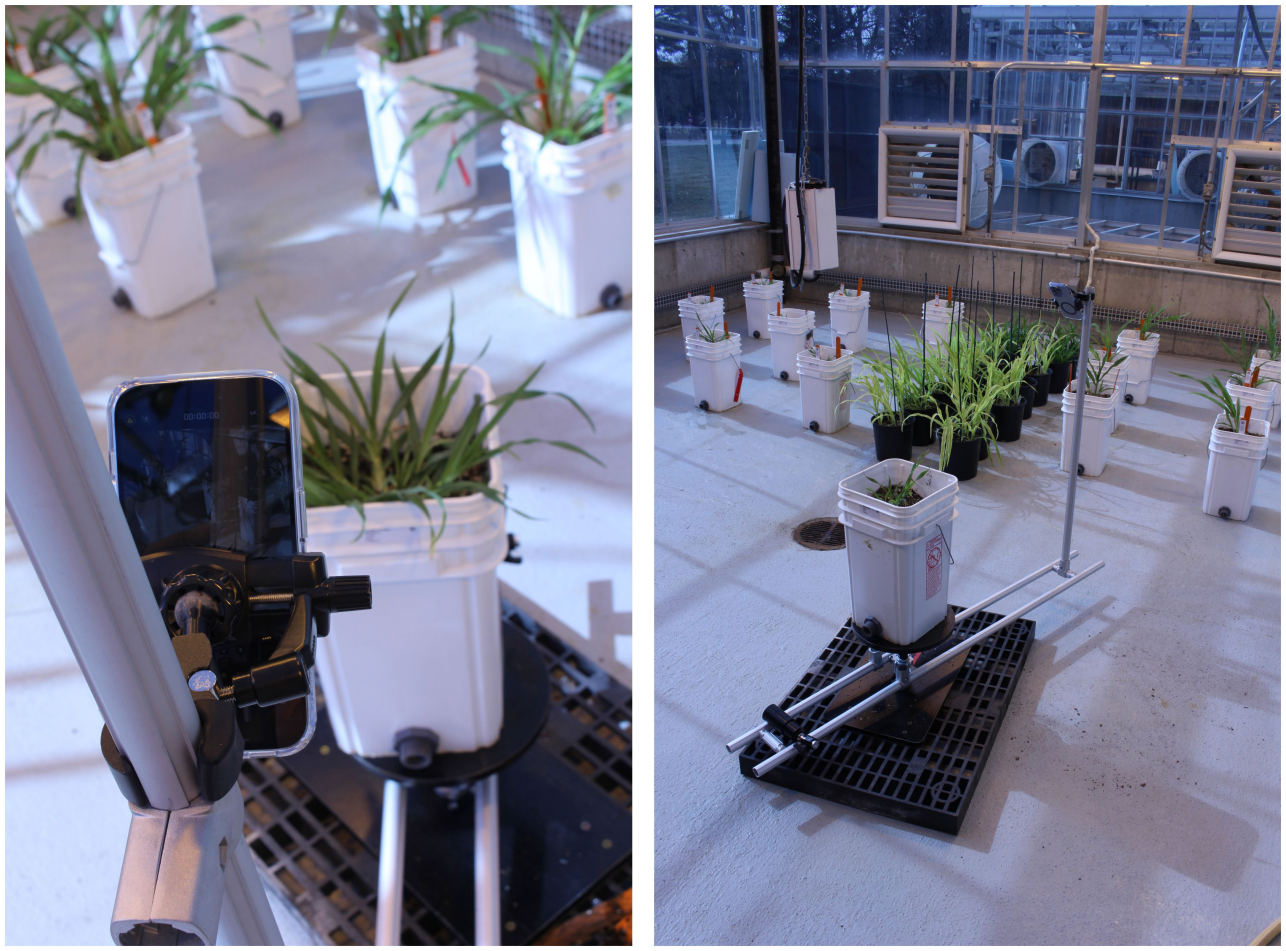
Imaging platform used in 2024 for 3D reconstruction. An iPhone 14 Pro, was mounted on a rotating turntable arm to capture comprehensive visual data.

To generate accurate 3-D models of finger millet plants at each phenological stage, we used an iPhone 14 Pro (48MP, 4K video recording, LiDAR Scanner). Two complementary capture methods were employed. First, the Polycam application was used to capture 25–50 high-resolution images per plant, with the device moved around each plant at varying heights and distances to sample diverse viewpoints. Polycam leverages the iPhone’s LiDAR sensor for real-time pose estimation, providing immediate feedback on viewpoint coverage during acquisition. Second, high-resolution video (4K, 60 fps) was recorded using a RAUBAY 360° spinner turntable from two vertical positions: mid-level and overhead, to ensure comprehensive structural coverage, particularly for taller plants at advanced growth stages (**Figure 1**). Videos were subsequently converted into image sequences using FFmpeg at 4 frames per second, yielding approximately 80–120 images per rotation. Each plant was placed individually on the turntable, and reference markers were included in the scene to support feature detection and color correction during post-processing. It is worth noting that the iPhone 14 Pro captures RGB imagery and LiDAR depth data from physically offset sensor positions, which can introduce parallax and occlusion effects when combining these modalities — a recognized challenge in multimodal plant imaging (Stumpe et al., 2025).

### 2.5 Neural radiance fields and virtual reality

Following image acquisition, camera pose estimation was performed using GLOMAP (Pan et al., 2024), a globally optimized Structure-from-Motion framework built upon COLMAP (Schönberger et al., 2016) that completed pose estimation in under 10 minutes per dataset, compared to 40–90 minutes for standard COLMAP workflows, while providing improved global alignment stability in the low-texture greenhouse environment. The resulting image sets and camera poses were then fed into the Nerfacto configuration within Nerfstudio (Tancik and Weber, 2023) for 3-D reconstruction. Nerfacto was selected for its multi-scale rendering and adaptive proposal sampling, which allocates compute toward structurally complex regions while simplifying low-detail areas, making it well-suited for capturing the architectural complexity of finger millet across growth stages (Arshad et al., 2024; Hu et al., 2024). Training was performed with fixed 30, 000 iterations, an exponentially decaying learning rate (1 × 10^-2^ → 1 × 10^-4^), a batch size of 4, 096 rays, 256 + 96 proposal network samples, and 48 final network samples, requiring approximately 20 minutes per model on an NVIDIA GeForce RTX 3090 GPU (24 GB VRAM) with an Intel Core i9 CPU and 128 GB RAM.

Within the broader landscape of 3-D reconstruction methods in agriculture, NeRF occupies a distinct niche: compared to classical Structure-from-Motion pipelines, which produce sparse point clouds less suited for immersive display, NeRF generates photorealistic volumetric renders that are particularly well-suited for visualization and training applications (Li et al., 2025; Clini et al., 2024). A known limitation of NeRF for plant applications is reduced fidelity on thin or semi-transparent structures such as fine leaves and stems, where volumetric smoothing can introduce blurring artifacts, particularly apparent in early vegetative stages with sparse canopy structure (Li et al., 2025; Hu et al., 2024).

Post-processing was performed in CloudCompare (2024) in two steps. First, statistical outlier removal (SOR) was applied after manual removal of background elements such as pots: a nearest-neighbor count of 50 with a standard deviation multiplier of 2 was used for aggressive noise reduction in dense canopy regions, while a count of 20 with a multiplier of 1 was applied where finer structural detail required preservation. Second, color correction was applied via a global RGB shift, sampling a target color from a 2-D reference image and applying the offset uniformly across the point cloud. Adjusted point clouds were exported in LAS format with 16-bit color encoding and subsequently imported into Unreal Engine (Epic Games) for integration into an immersive virtual greenhouse environment navigable via Meta Quest 2 VR headsets (Meta Platforms Inc.). This virtual environment allows breeders and researchers to inspect plants at each phenological stage from multiple perspectives without physical presence, supporting both standardization of growth stage training and reduction of observer bias across raters (Sarkar et al., 2023; Ganapathysubramanian et al., 2025).

## 3 Results

The BBCH scale was applied to finger millet to document key phenological stages relevant to breeding and crop management. Observations were organized according to the main growth stages, with emphasis on those most critical for staging and imaging.

### 3.1 Germination: growth stage 0

**Figure 2:**
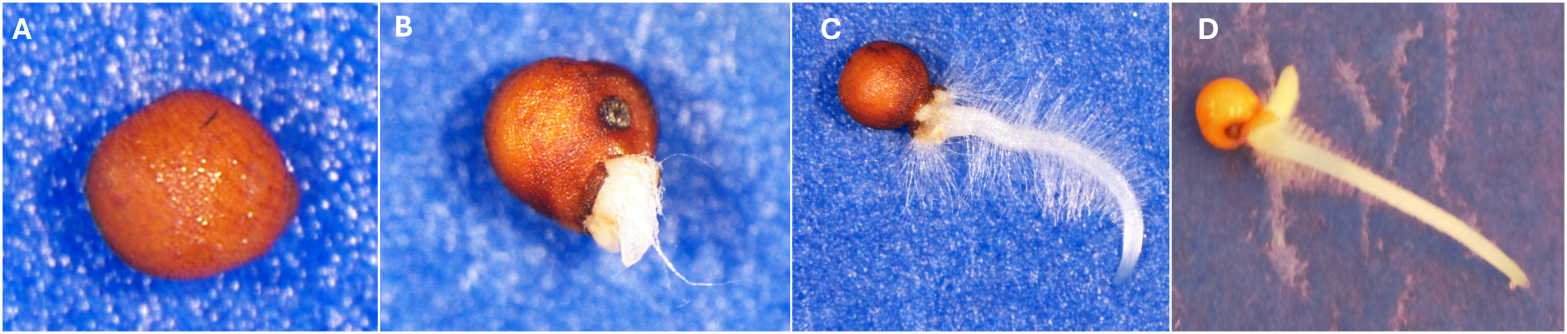
Stages of finger millet seed germination according to BBCH scale. **(A)** Seed imbibition (BBCH 03): the caryopsis has absorbed water and become visibly swollen. **(B)** Radicle emergence (BBCH 05): the radicle breaks through the seed coat. **(C)** Radicle elongation (BBCH 06): the radicle continues to grow and extend. **(D)** Coleoptile emergence (BBCH 07): the coleoptile begins to protrude from the caryopsis marking the onset of shoot development.

Germination begins with the imbibition of caryopsis (BBCH 01) and concludes after the coleoptile emerges from the soil surface (BBCH 09). The most critical substages are described in (**Figure 2**). Initially, the caryopsis absorbs water and rehydrates its tissues by imbibition until it is swollen (BBCH 03) (**Figure 2A**).

This is followed by radicle emergence of the caryopsis (BBCH 05) (**Figure 2B**), where the radicle sticks out from the caryopsis, and begin elongation. The development of this stage requires approximately 14.5 °C d. Subsequent stages include primary root elongation (BBCH 06) (**Figure 2C**), and coleoptile emergence (BBCH 07) (**Figure 2D**), which protects the developing primary leaves until they break through the soil surface at an accumulated thermal time of 43.5 °C d.

### 3.2 Leaf development: growth stage 1

**Figure 3:**
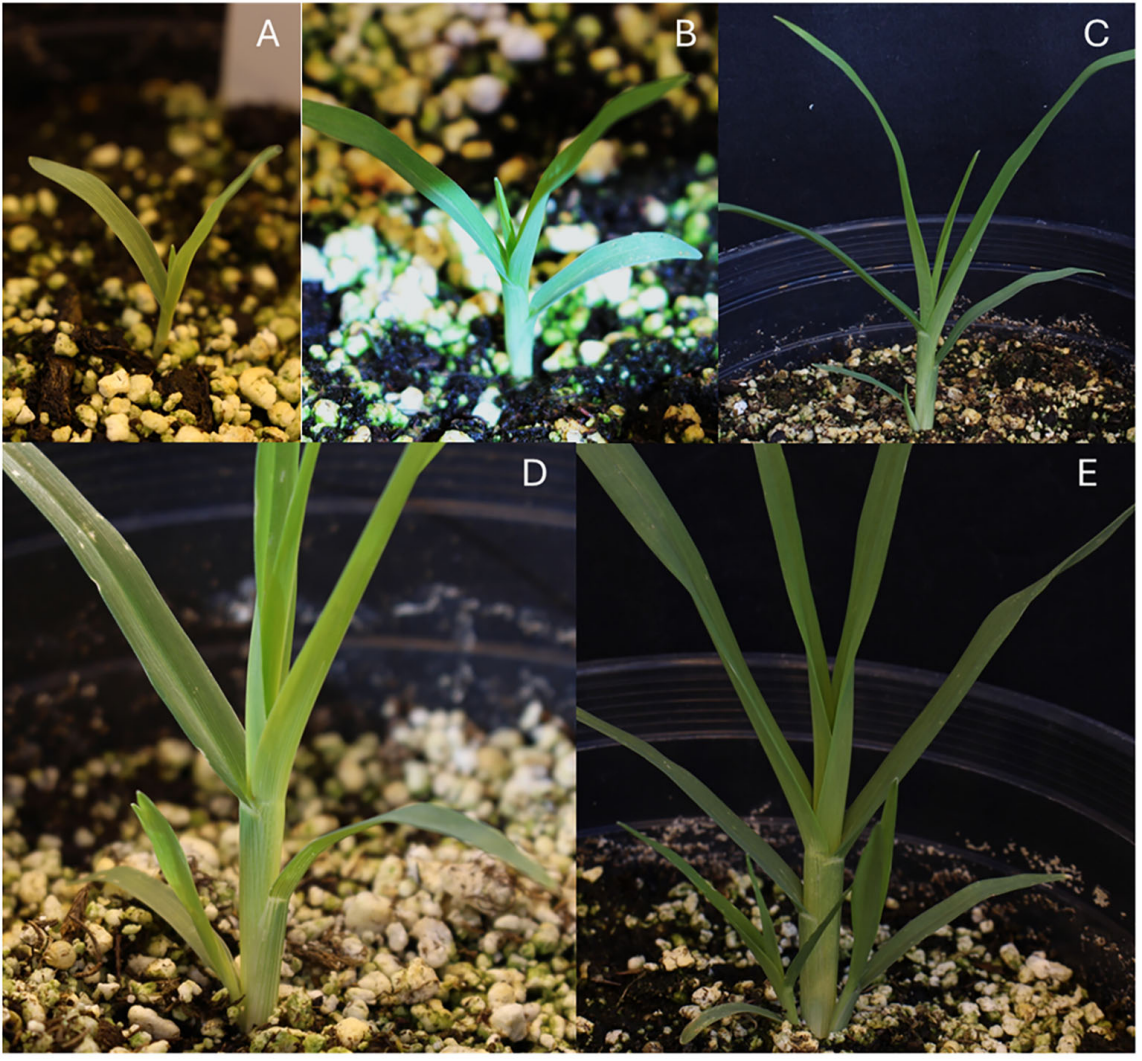
Vegetative growth stages of finger millet, according to BBCH scale. **(A)** BBCH 11, Emergence of the first leaf with visible collar. **(B)** BBCH 12: Second leaf with visible collar; leaves begin to alternate through the pseudo stem. **(C)** BBCH 13: Third leaf fully expanded; simultaneously the first tiller start to develop in the base of the stem. **(D)** BBCH 14: Fourth leaf expanded, first tiller continues to develop. **(E)** BBCH 15: Fifth true leaf expanded, and the second tiller begins to develop (BBCH 22).

GS 1 marks the emergence of leaves on the main stem. Leaves appear individually in an alternate pattern, extending from the stem at different angles and displaying a prominent midrib. This stage tracks the number fully expanded leaves on the main stem, defined by the presence of a visible collar in the neck of the leaf blade. The first sub-stage begins with the emergence of the first leaf from the coleoptile (BBCH 10) at approximately 5 days after planting (DAP), and an accumulated thermal time of 73 °C d. Once the collar on the first leaf is visible, the stage is coded as BBCH 11 (**Figure 3A**). Leaf emergence continues in an alternating pattern along the stem (**Figures 3B, C**). The visible collar marker reduces the bias of observers and aligns with established scales in maize. Its reproducibility across genotypes and environments makes it valuable for breeding purposes, where tracking vegetative development across different environments is essential.

### 3.3 Tillering: growth stage 2

GS 2 describes the development of side shoots, or tillers, from the base of the stem. A tiller was considered visible when its length reached one centimeter. The first tiller (BBCH 21) was observed when three to four leaves have expanded in the main stem (**Figure 3D**). Second tiller (BBCH 22) was observed at approximately 319 °Cd. (**Figure 3E**).

The number of tillers varies depending on genotype and environmental conditions, and in some instances, plants produced more than ten tillers. While the number of tillers varies, this trait provides an indication of plant vigor and resource allocation. Because some tillers differentiate into panicle and flowers, while others remain vegetative at maturity, it is a relevant feature that offers insights into yield components and stress responses.

### 3.4 Stem elongation: growth stage 3

GS 3 encompass stem elongation. In this study, all four accessions exhibited erect stems, although other genotypes may display decumbent or prostrate growth types, which were not included in this study. The stem is typically smooth, slightly flattened, and green, with some genotypes occasionally displaying purple pigmentation at the nodes.

The node count varies from five to seven across genotypes. The first node was detected when at least twenty leaves had emerged on the main stem. From the base of the stem upwards, nodes are detected by sliding the fingers through the stem. Since the leaf sheath protects the nodes, gentle physical pressure was exerted on the stem with fingers to identify them. Although this stage was recorded for practical staging purposes, it was excluded from the proposed scale due to the difficulty in consistency to locate the first node in the plant. This exclusion reflects a methodological choice aimed at prioritizing stages that are both agronomically relevant and straightforward to identify, thereby enhancing reproducibility and reducing observer bias in field applications.

### 3.5 Heading (inflorescence emerging): growth stage 5

**Figure 4:**
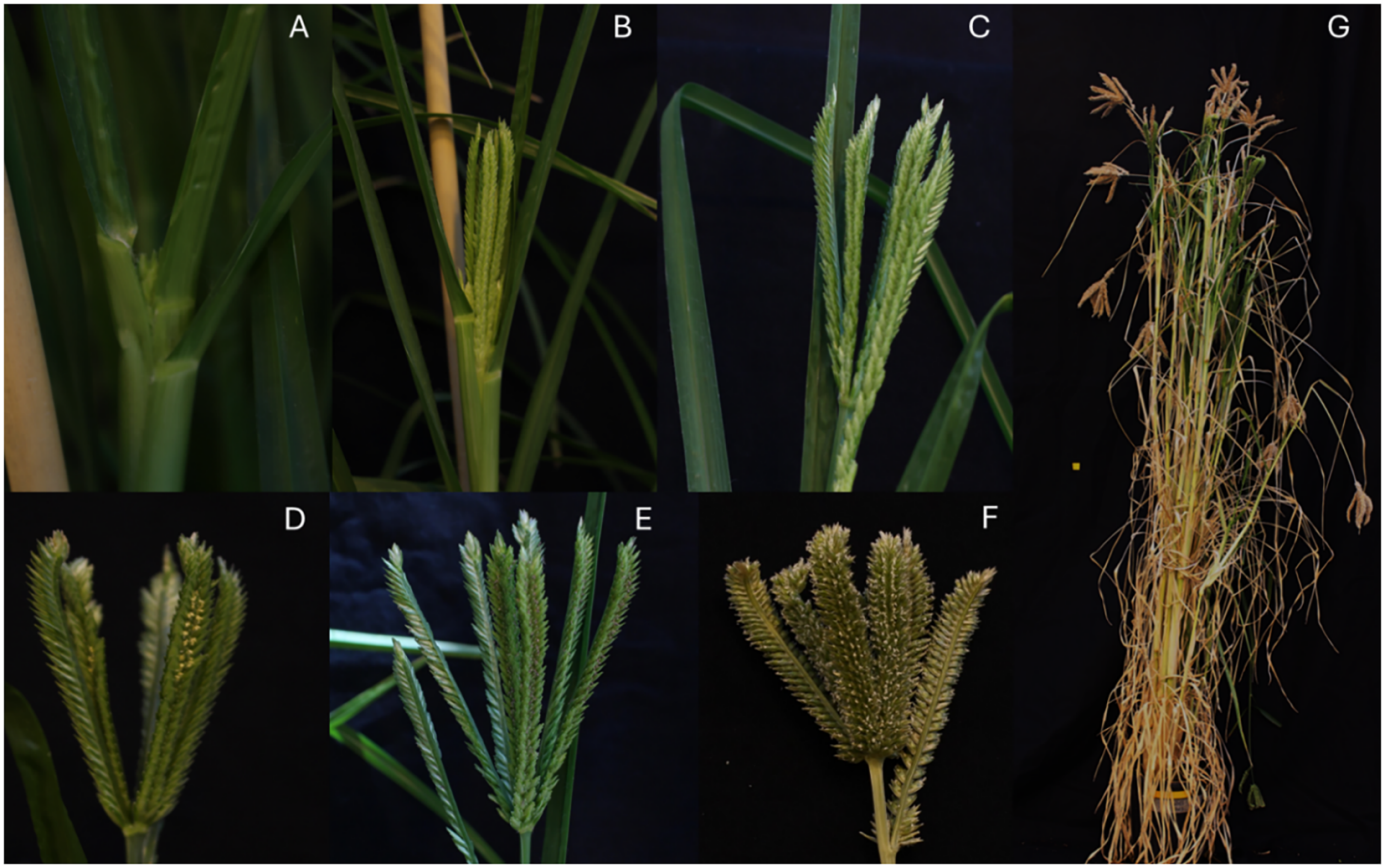
Reproductive growth stages of finger millet, according to BBCH scale. **(A)** BBCH 51: Initiation of head emergence; the inflorescence begins to emerge from the last visible leaf (flag leaf). **(B)** BBCH 55: Partial emergence; approximately 50% of the head is visible. **(C)** BBCH 59: Full head emergence; no anthers are yet visible. **(D)** BBCH 61: Onset of flowering; anthers first appear at the top of the fingers. **(E)** BBCH 65: Mid-flowering; anthers are visible on approximately half portion of the fingers. **(F)** BBCH 69: Completion of flowering; anthers are now visible at the base of the fingers. **(G)** BBCH 95: Senescence stage; around 80% of the foliage has turned brown, indicating physiological maturity.

The heading stage marks the emergence of inflorescence through the main stem. It begins with BBCH 5, when the tip of the fingers (panicle) becomes visible through the flag leaf (**Figure 4A**). This stage occurs approximately 71 DAP and when the plant reach an accumulated thermal time of 1030 °C d. As stem elongation continues, the panicle progressively emerges. Further substages represent the percentage of panicle emergence through the stem. BBCH 55, represents approximately 50% panicle emergence (**Figure 4B**), and BBCH 59 indicates fully emergence of the panicle from the flag leaf sheath (**Figure 4C**). These stages are useful for tracking the timing and extent of inflorescence development.

### 3.6 Flowering: growth stage 6

**Figure 5:**
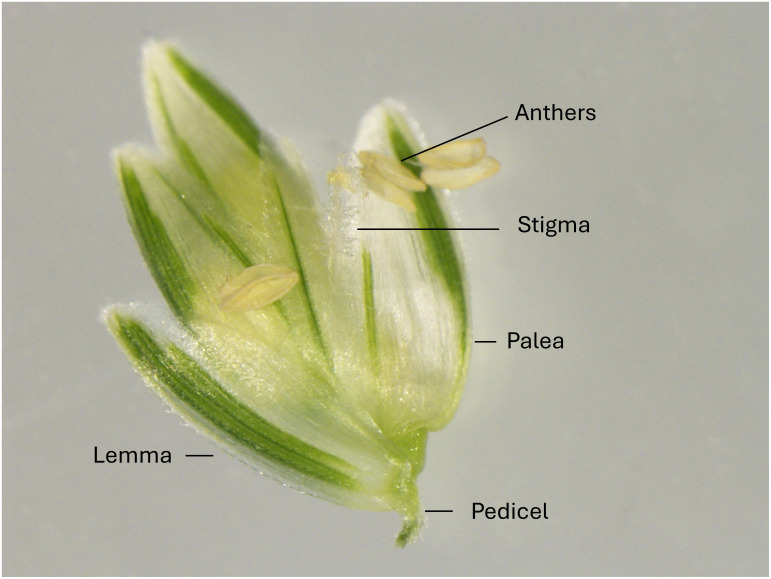
Floral anatomy of finger millet. Anthers become visible following pollination and exhibit a genotype dependent coloration, ranging from yellow and creamy to different shades of purple.

Finger millet is predominantly self-pollinated, with pollination occurring prior to the visible opening of the flowers. Flowering is defined by the appearance of anthers outside the florets. In finger millet, anthers are typically purple or cream-colored and easily identifiable (**Figure 5**). Flowering initiates at the tip of the fingers and progresses towards the base in a basipetal pattern. Secondary substages quantify the proportion of the finger length with visible anthers (**Figures 4D–F**). This morphological sequence provides a robust and reproducible marker for staging, reducing observer subjectivity. Its consistency across genotypes and environments makes it particularly valuable in breeding programs, where precise timing of flowering events are critical for crossing, stress screening, and yield evaluation.

Flowering typically spans 5 to 7 days and is influenced by environmental conditions. Flowering was completed when the anthers dehydrate and fall off, making this stage susceptible to the timing of observation and prevailing environmental conditions. It is not uncommon for anthers to be visible before the head is fully expanded throughout the last leaf. In such cases, overlapping substages from GS5 and GS6 may be used to accurately describe both events.

### 3.7 Seed development: growth stage 7

**Figure 6:**
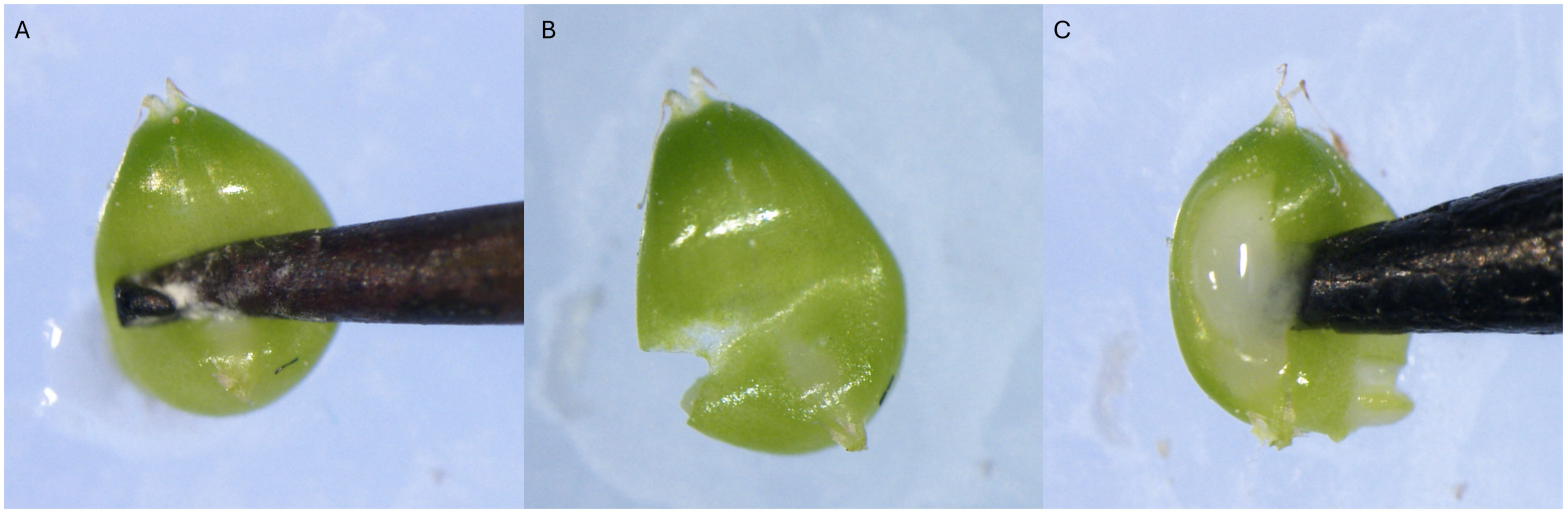
Seed development stages of finger millet according to BBCH scale. **(A, B)** BBCH 71: Early grain filling stage; the seed content is a translucent aqueous liquid. **(C)** BBCH 75: Mid grain filling stage; the seed content transitions to a milky, viscous fluid.

Seed development in finger millet follows a three-phase pattern, which is common to many cereal crops (Adams and Rinne, 1980). Two key substages were observed in this phase. BBCH 71, describes a seed containing aqueous fluid evidenced by the excretion of a translucent liquid when gently squeezed (**Figures 6A, B**) which takes about 84 DAP to stage, with an accumulated thermal time of 1218 °C d. The next stage is characterized by starch accumulation in the seed and corresponds to the BBCH substage (75). It is described as a seed with white sticky fluid when the seed is gently squeezed (**Figure 6C**) occurring around 86 DAP and when the plants reached 1247 °C d. These substages (71-75) correspond to phase II of seed development, which starts with the dry matter accumulation in the seed, initially by increasing the moisture content in the seed and later by replacing accumulated water with starch (Adams and Rinne, 1980). While these stages provide agronomically meaningful markers of maturity, their accurate determination requires seed dissection, which is challenging in small-seeded cereals. This methodological limitation underscores the need for careful handling but also highlights the scale’s robustness in capturing critical transitions toward harvest maturity.

### 3.8 Ripening: growth stage 8

**Figure 7:**
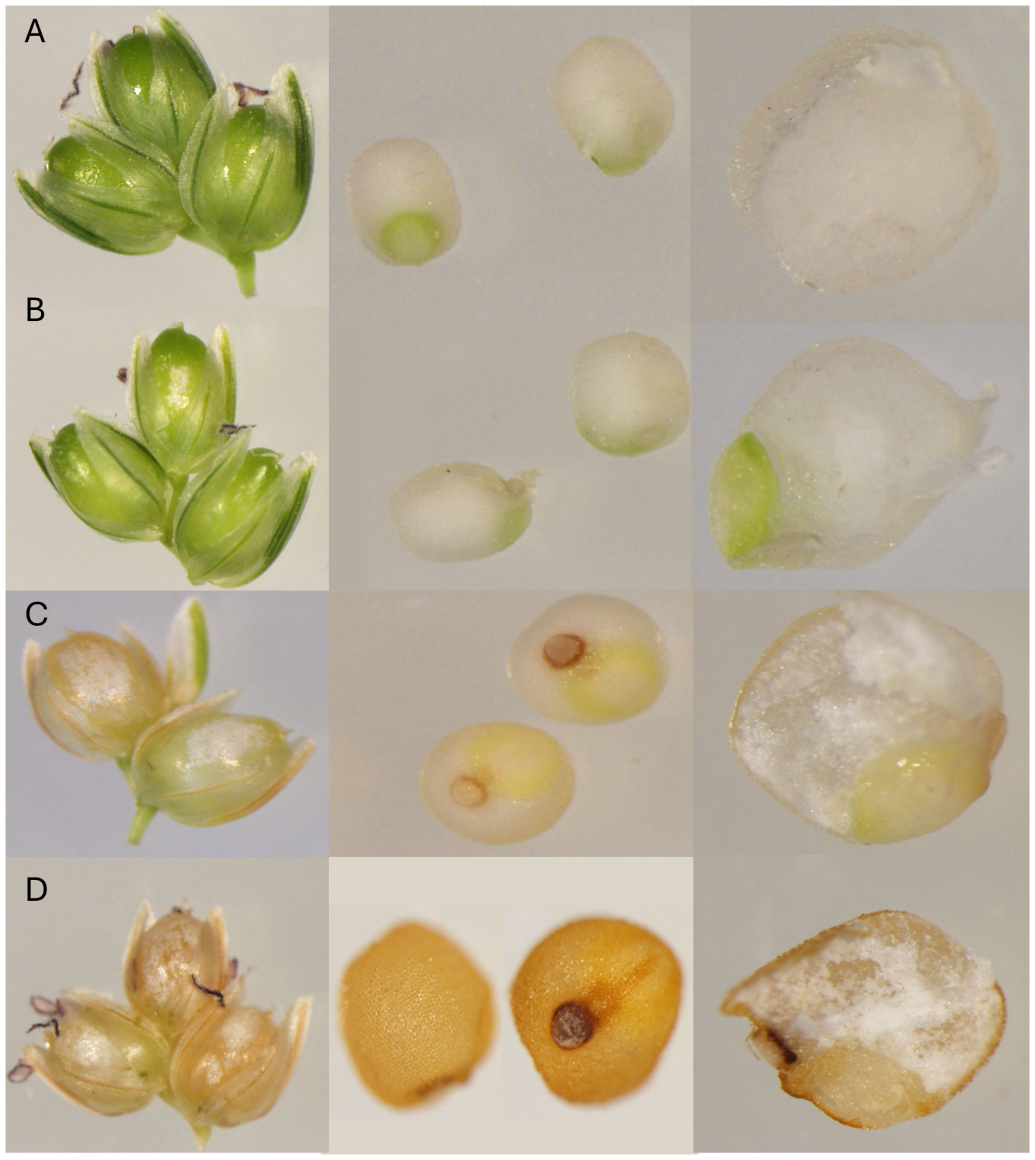
Seed ripening stages of finger millet according to BBCH scale. **(A)** BBCH 83: Early dough stage; when the seed is divided, the content of it is still wet. **(B)** BBCH 85: Soft dough stage; the content of the seed is dry and soft. **(C)** BBCH 87: Hard dough stage; the content of the seed is dry and hard. **(D)** BBCH 92: Physiological maturity; the seed is over-ripe and a distinct black layer is visible, indicating full maturity of the seed.

Ripening is categorized into three substages based on the hardness of the seed content. The first substage (BBCH 83) is an early dough stage, where the seed is soft and moist when split with a fingernail (**Figure 7A**). This stage requires an accumulated thermal time of 1320 °C d, at approximately 91 DAP. As the seeds progress toward maturity, their content gets harder and drier. BBCH 85 is characterized by a seed soft and dry (**Figure 7B**). Finally, the last substage consists of a hard and dry seed content (BBCH 87). Externally, the coloration of the glumes enclosing the seed turns yellow as the plant matures (**Figure 7C**).

### 3.9 Senescence: growth stage 9

Senescence begins when the caryopsis becomes hard and cannot be split with the fingernail. At this stage, seed coats exhibit distinct coloration (white, light brown, dark brown, copper brown). Physiological maturity is marked by maximum seed dry weight and the appearance of a black layer at the base of the seed (BBCH 92) (**Figure 7D**). This stage requires an accumulated thermal time of 1537 °C d, approximately 106 DAP. Dry down is remaining for the seed as well as the leaves and stem. Final senescence (BBCH 95), is reached when more than 50% of the plant has dried, indicated by a brown coloration on the plant organs, and loss of turgor (**Figure 4G**). As plants progress to final senescence, with widespread tissue desiccation and loss of turgor, the scale captures the transition to harvest readiness, ensuring consistency in maturity assessment across environments.

## 4 Discussion

To support breeders and researchers, we propose a simplified phenological scale for finger millet based on the most relevant and easily identifiable stages observed in this study (**Table 1**). The scale divides the growth cycle into two principal stages: vegetative and reproductive. The vegetative phase encompasses leaf and tiller development, while the reproductive phase includes heading, flowering, seed filling, ripening, and senescence. Consistent with the BBCH framework, all stages and substages were recorded on the main stem. Beyond providing descriptive clarity, this framework is intended to facilitate standardization across environments and genotypes, ensuring that researchers can reliably compare developmental data and integrate findings into breeding pipelines. By focusing on stages that are both agronomically relevant and easy to identify, the scale reduces observer bias, improves reproducibility, and strengthens its value for breeding programs and phenological research.

**Table 1:** Comparison of the BBCH scale and the proposed phenological scale for Finger millet, highlighting Vegetative, Heading, and Reproductive stages.

Code BBCH	Proposed Scale	Description	Days to stage	GDD (°CDay)	Figure
00		3.1 Germination Section:			
03		Seed imbibition finished			2.A
05		Radicle emerging from caryopsis	1	14.5	2.B
06		Radicle elongated			2.C
07		Coleoptile emerging from caryopsis	3	43.5	2.D
10		3.2 Leaf development Section:			
11	V1	First leaf expanded	5	73	3.A
12	V2	Second leaf expanded	12	174	3.B
13	V3	Third leaf expanded	15	218	3.C
1n	Vn	n leaf expanded			
20		3.3 Tillering Section:			
21		First visible tiller in the base of stem			3.D
22		Second visible tiller in the base of stem	22	319	3.E
30		3.4 Stem elongation Section:			
31		First node detected on the base of stem			
32		Second node detected			
33		Third node detected			
50		3.5 Heading Section:			
51	H1	Tip of panicle visible through the last leaf	71	1030	4.A
55	H2	Panicle 50% emerged through the stem			4.B
59	H3	Heading completed: All fingers have emerged through the sheath			4.C
60		3.6 Flowering Section:			
61	R1	Start of flowering. Anthers visible in the tip of fingers			4.D
65	R2	Full flowering. 50% of anthers visible in the inflorescence of main stem	77	1117	4.E
69	R3	End of flowering. Anthers are visible at the bottom of fingers. Anthers in the tip are dry or senesced.			4.F
70		3.7 Seed development Section:			
71	R4	Blister. Grains start forming. The content of the grain is an aqueous fluid	84	1218	6.A-B
75	R5	Milky grain. Grains achieved maximum size. The content of the grain is a milky fluid	86	1247	6.C
80		3.8 Ripening Section:			
83	R6	Early Dough	91	1320	7.A
85	R7	Soft Dough	94	1363	7.B
87	R8	Hard Dough			7.C
90		3.9 Senescence Section:			
92	R9	Over-ripe. Grain very hard. Physiological maturity, black layer formation	106	1537	7.D
95	R10	>50% of canopy senesced or dry	114	1653	4.G

Stage durations represent the average number of days across four accessions. Growing degree days (GDD) were calculated using mean daily greenhouse temperatures with a base temperature of 10 °C.

Vegetative stages focus on leaf development. Each stage is denoted by the prefix V, followed by the number of fully expanded leaves with visible collars. This approach is very similar to the vegetative stages described for maize (Hanway, 1966). The final number of leaves varies by genotype and is represented by Vn, where n is the total count of fully expanded leaves on the main stem. This notation provides a straightforward method for tracking vegetative progression across different cultivars and environments. As a limitation, stem elongation stages were not included in the simplified scale. Accurate detection of nodes on the main stem requires a highly skilled observer, and progression through these stages occurs rapidly. Moreover, since the most relevant developmental transition after leaf expansion is the emergence of the inflorescence, this latter stage was considered the next critical milestone in the simplified framework.

The reproductive phase in finger millet begins with the heading stages, denoted by the prefix “H, ” which mark the emergence of the inflorescence from the leaf sheath. This phase includes two substages: H1, indicating the initial appearance of the inflorescence, and H5, representing the point at which approximately 50% of the inflorescence has emerged (**Table 1**). Following heading, the crop enters the flowering, seed development, ripening, and senescence stages, which are collectively referred to as reproductive stages and are denoted by the prefix “R.”

Flowering is captured in the first three reproductive substages (R1-R3) which describe the visibility of anthers along the panicle. R1 marks the appearance of anthers at the tip of the fingers. As flowering in finger millet progresses in a basipetal pattern, R2 corresponds to anthers visible in the middle of the fingers, coinciding with approximately half of the flowering process completed. R3 marks the end of flowering, with anthers visible at the base of the fingers. At this stage, anthers at the tip and middle may show signs of dehydration and color change.

This staging framework provides a reliable and reproducible method for tracking reproductive progression, as the clear morphological markers (anther visibility and position) minimize observer subjectivity and ensure consistency across environments and genotypes.

Seed development begins after flowering, starting with R4, when the caryopsis contains aqueous fluid, that can be observed by gently squeezing the seed. This fluid is gradually replaced by starch, and stage R5 is characteristic of a milky, sticky fluid within the caryopsis. These stages are not easily observed and require manual inspection by removing see from the central portion of a finger and compressing it between fingernails.

As the grain matures, moisture content decreases, leading to three distinct ripening stages. R6 represents the early dough stage, where the grain content is soft and wet. R7 marks the soft dough stage, with grain content that is soft but dry. R8 indicates the hard dough stage, where grain becomes firm and dry. A limitation of this staging framework is that accurate determination requires seed dissection, which can be particularly challenging in small-seeded cereals such as finger millet. This methodological constraint may limit the ease of application in routine field evaluations, as specialized handling and careful observation are necessary to reliably distinguish between the dough stages.

Physiological maturity is reached at stage R9, identified by a hard caryopsis that is difficult to split with a fingernail and has a dark hilum (black layer), indicating that vascular tissue supplying assimilates to the seed has ceased to function. The final stage R10 denotes the harvest maturity, when at least 50% of the plant (foliage, stem, inflorescence) has a dry brown coloration.

We complemented growth staging and 2-D imaging with 3-dimensional rendering at each phenological stage to leverage advances in imaging modalities in the age of phenomics and AI (Arshad et al., 2024; Atkinson et al., 2019; Singh et al., 2021c; Xiang et al., 2020). The resolution of the modeled plants was defined by the total number of points in each point cloud, ranging from 2.4 million points at early vegetative stages to 74.6 million points at later reproductive stages, reflecting the progressive increase in canopy biomass and structural complexity across development. This scaling of point cloud density with growth stage is agronomically meaningful, as denser reconstructions at reproductive stages captured finer morphological detail relevant to heading, flowering, and seed development assessment.

While useful for visualization purposes, creating these 3-D models demanded considerable computing and time investment: data collection took approximately 5–7 minutes per plant; data processing 30–60 minutes; model training ~20 minutes; point cloud export ~30 minutes; and cleaning, color adjustment, and virtual environment setup an additional 3–4 hours. Unlike traditional 2-D imaging, which captures plant morphology from a fixed perspective, NeRF-based reconstruction enables simultaneous inspection of the same plant from any viewpoint and at multiple developmental stages within a single virtual environment, reducing observer bias and supporting more consistent cross-environment staging (Li et al., 2025; Clini et al., 2024; Hu et al., 2024). This virtual environment provided an interactive platform for breeders and researchers to explore growth stages and phenological traits in a lifelike setting (Sarkar et al., 2023; Ganapathysubramanian et al., 2025). The 3-D phenotyping methods have shown utility in stress and canopy trait assessment (Chiranjeevi et al., 2021; Young et al., 2023), and to our knowledge this is the first application of NeRF-based 3-D imaging to accompany a phenological staging scale in any underutilized cereal crop. In the future, there will be tremendous value in including 3-D imaging to complement BBCH charts in major crops, as it will allow easier and more accurate training of new researchers as well as standardization of protocols and removal of phenotyping biases across raters.

**Figure 8:**
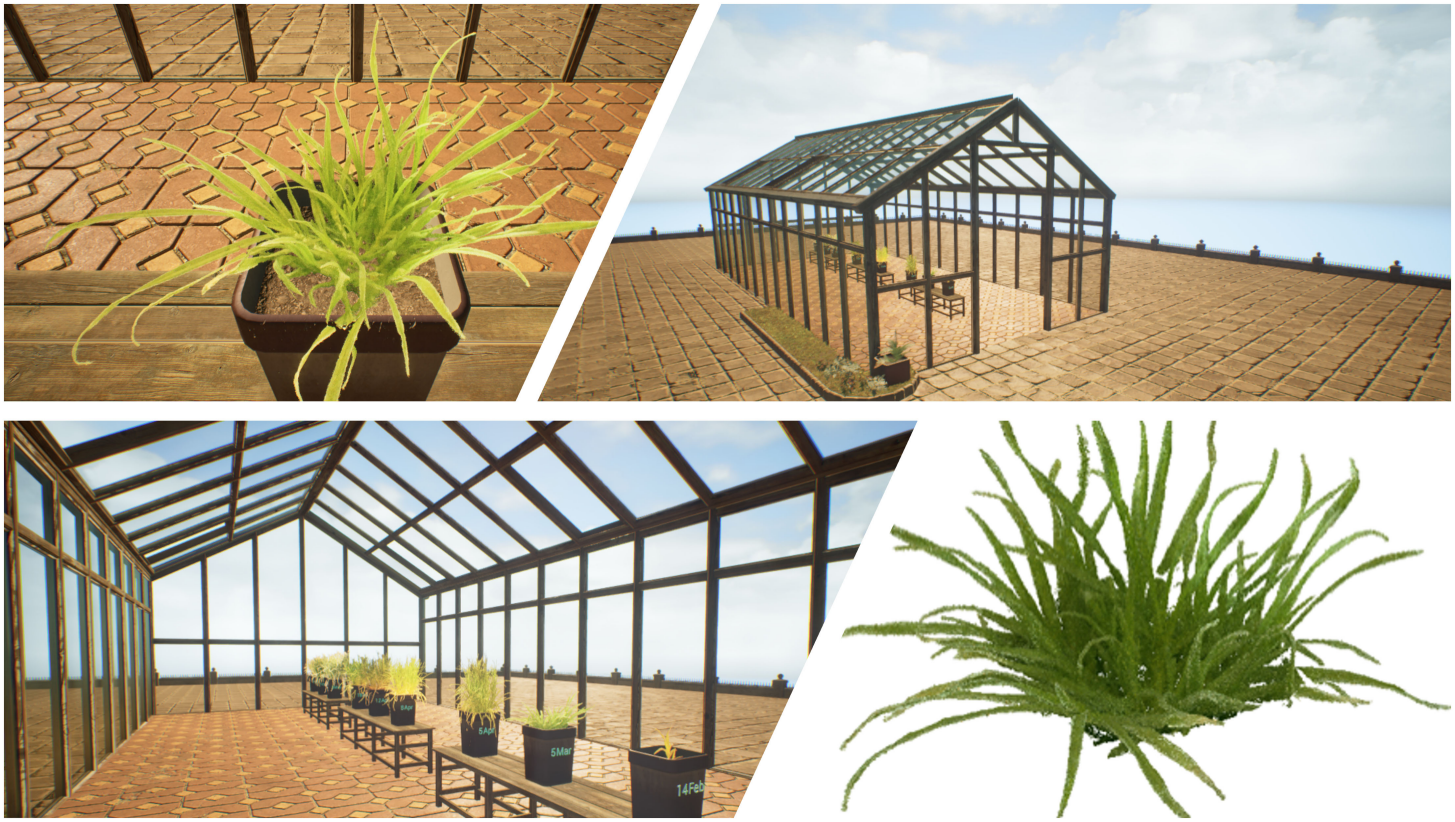
Example of a 3D model reconstruction within a virtual greenhouse environment. Each bucket placed on the benches represents a specific date of phenological stage. The interactive interface allows users to zoom in, navigate around the space, and examine individual plants from multiple perspectives.

The cleaned 3-D models were integrated into a virtual reality environment using Unreal Engine, where users can wear VR headsets (Meta Quest 2, Meta Platforms Inc.) to explore the greenhouse, inspect plants at each growth stage, and observe morphological progression without being physically present (**Figure 8**).

## 5 Conclusion

Finger millet has received minimal resources compared to other cereal crops such as maize, wheat, rice, and sorghum, which hinders its breeding and research efforts. However, its nutritional value, hardiness, and economic potential is attracting global interest from farmers and industry. As a first step for the needs of research standardization, we used the BBCH chart as the baseline for describing the main developmental stages in Finger millet species and document them visually. Additionally, we provide a simplified and user-friendly phenological growth staging scale that allows researchers and breeders to standardize the stages and compare different cultivars and environments. Finally, for ease of understanding and training, we show the usefulness of the NeRF method to develop 3-D rendering to align them with growth stages. It is the first report on the use of 3-D imaging to present growth and development staging.

## Data Availability

The raw data supporting the conclusions of this article will be made available by the authors, without undue reservation.
